# Sex differences in uterine artery Doppler during gestation in pregnancies complicated by placental dysfunction

**DOI:** 10.1186/s13293-021-00362-7

**Published:** 2021-02-02

**Authors:** Leah Paranavitana, Melissa Walker, Anjana Ravi Chandran, Natasha Milligan, Shiri Shinar, Clare L. Whitehead, Sebastian R. Hobson, Lena Serghides, W. Tony Parks, Ahmet A. Baschat, Christopher K. Macgowan, John G. Sled, John C. Kingdom, Lindsay S. Cahill

**Affiliations:** 1 Department of Chemistry, Memorial University of Newfoundland, 283 Prince Philip Drive, St John’s, Newfoundland and Labrador A1B 3X7 Canada; 2 Mount Sinai Hospital, Toronto, Ontario Canada; 3 Department of Obstetrics and Gynecology, University of Toronto, Toronto, Ontario Canada; 4 Division of Cardiology, Department of Paediatrics, The Hospital for Sick Children, Toronto, Ontario Canada; 5 Pregnancy Research Centre, Department of Obstetrics and Gynaecology, Royal Women’s Hospital, Parkville, Australia; 6 Toronto General Hospital Research Institute, University Health Network, Toronto, Ontario Canada; 7 Department of Immunology and Institute of Medical Sciences, University of Toronto, Toronto, Ontario Canada; 8 Women’s College Research Institute, Women’s College Hospital, Toronto, Ontario Canada; 9 Department of Pathology, Mount Sinai Hospital, Toronto, Ontario Canada; 10 Department of Laboratory Medicine and Pathobiology, University of Toronto, Toronto, Ontario Canada; 11 Centre for Fetal Therapy, Johns Hopkins Medicine, Baltimore, Maryland USA; 12 Translational Medicine, The Hospital for Sick Children, Toronto, Ontario Canada; 13 Department of Medical Biophysics, University of Toronto, Toronto, Ontario Canada; 14 Mouse Imaging Centre, The Hospital for Sick Children, Toronto, Ontario Canada

**Keywords:** Doppler ultrasound, Fetal sex, Pregnancy, Pulsatility index, Sex differences, Uterine artery

## Abstract

**Background:**

There is growing evidence of sex differences in placental vascular development. The objective of this study was to investigate the effect of fetal sex on uterine artery pulsatility index (PI) throughout gestation in a cohort of normal and complicated pregnancies.

**Methods:**

A prospective longitudinal study was conducted in 240 pregnant women. Pulsed wave Doppler ultrasound of the proximal uterine arteries was performed at a 4-weekly interval between 14 and 40 weeks of gestation. The patients were classified retrospectively as normal or complicated (one or more of maternal preeclampsia, preterm birth, or small for gestational age). To assess if the change in uterine artery PI during gestation differed between normal and complicated pregnancies and between fetal sexes, the uterine artery PI was modeled using a linear function of gestational age and the rate of change was estimated from the slope.

**Results:**

While the uterine artery PI did not differ over gestation between females and males for normal pregnancies, the trajectory of this index differed by fetal sex for pregnancies complicated by either preeclampsia, preterm birth, or fetal growth restriction (*p* < 0.0001). The male fetuses in the complicated pregnancy group had an elevated slope compared to the other groups (*p* < 0.0001), suggesting a more progressive deterioration in uteroplacental perfusion over gestation.

**Conclusions:**

The uterine artery PI is widely used to assess uteroplacental function in clinical settings. The observation that this metric changes more rapidly in complicated pregnancies where the fetus was male highlights the importance of sex when interpreting hemodynamic markers of placental maturation.

## Background

Metabolic demand increases exponentially as gestation progresses, thereby requiring that the uteroplacental circulation adapt to deliver increased blood flow. Insufficient uterine vascular remodeling is associated with preeclampsia, preterm birth, and fetal growth restriction [1–3]. Clinically, changes in uteroplacental blood flow are measured by uterine artery Doppler waveforms and described by the pulsatility index (PI). In healthy pregnancies, the uterine artery PI decreases over gestation [4, 5]. An increased uterine artery PI, with or without an early diastolic notch, is an indicator of uteroplacental vascular insufficiency, and has been demonstrated in cross-sectional studies to be associated with poor perinatal outcomes [6–9].

Sex-specific differences in pregnancy outcomes are well-established. For example, male fetuses are at an increased risk of fetal distress, preterm birth, or early neonatal death [10–14] while female fetuses have a greater risk of mortality during gestation [15]. There is a higher incidence of early onset preeclampsia in women carrying female fetuses [16–18]. Sex differences have also been reported for both placental structure and function [19–24]. Placental pathology has been shown to be dependent on fetal sex, with males exhibiting inflammatory pathology and females having a greater risk for placental infarction [25]. Recent studies have found sex differences in umbilical artery PI over the second half of gestation in uncomplicated pregnancies [26, 27]. In the present study, we investigated the effect of fetal sex on uterine artery PI throughout gestation in a cohort of normal and complicated pregnancies.

## Methods

### Patient cohort

Uterine artery measurements were acquired prospectively as part of a study that evaluated a new methodology for measuring umbilical artery hemodynamics. The latter study, which will be reported elsewhere, dictated the study sample size and inclusion criteria. A longitudinal ultrasound study was conducted in 240 women recruited in the first trimester of pregnancy. Inclusion criteria were maternal age between 18 and 45 years, body mass index (BMI) < 45 kg/m^2^, singleton pregnancy, and no significant maternal comorbidities such as type 1 diabetes or chronic hypertension. Datasets were excluded if the women withdrew at any point during the study or if the women delivered elsewhere and were lost to follow up. The study was approved by the Institutional Review Boards of The Hospital for Sick Children (Toronto, ON, Canada, REB Number 1000051548), Mount Sinai Hospital (Toronto, ON, Canada, REB Number 15-0279-A), and Johns Hopkins Hospital University (Baltimore, MD, USA, IRB Number 0082717).

### Ultrasound Doppler assessment

Ultrasound examinations were performed on a 4-weekly interval between 14 and 40 weeks of gestation by certified research sonographers using either a Philips iU22 (Philips Healthcare, Andover, MA, USA) or GE Voluson e10 (GE Healthcare, Chicago, IL, USA) ultrasound system. Pulsed Doppler spectra of the left and right proximal uterine arteries were collected at the crossover point of the external iliac artery and main uterine artery [28]. Patients were followed until delivery and pregnancies were classified retrospectively as either normal or complicated. Normal pregnancies were defined as normotensive women who delivered at term with neonatal birth weight appropriate for gestational age. Complicated pregnancies were defined as one or more of maternal preeclampsia (diagnosed according to ACOG guidelines [29]), preterm birth (delivery < 37 weeks’ gestation), or small for gestational age (SGA) neonate (birth weight < 10th centile according to population growth charts [30]). The PI for each uterine artery was computed from the traced average Doppler waveforms as the difference between the peak systolic (PSV) and end-diastolic velocities (EDV), divided by the time-averaged mean velocity (TAMV) over the cardiac cycle (PI = (PSV−EDV)/TAMV).

### Statistical analysis

All statistical tests were performed using the R statistical software package (www.r.project.org). Data are reported as mean ± standard deviation. The left and right uterine artery PI values were averaged to provide the overall mean PI. To analyze the clinical characteristics, a two-way ANOVA was used for continuous variables to evaluate the effect of group (normal, complicated) and fetal sex (female, male), and a Pearson’s chi-squared test was used for categorical variables. The uterine artery PI data was analyzed using a linear mixed effects model with gestational age (in completed weeks), group (normal, complicated), fetal sex (female, male), and race (Asian, Black or African American, White, Other) as the fixed effects and a heteroscedastic random effect where inter-subject variation varied linearly with gestational age. To assess if the change in uterine artery PI with gestational age was different between groups and fetal sex, the uterine artery PI was modeled as a linear function of gestational age and the change in uterine artery PI was estimated by the slope. A linear model was chosen based on previous studies of uterine artery PI throughout gestation [31, 32]. A value of *p* < 0.05 was taken to be significant.

## Results

### Patient characteristics

**Fig. 1:**
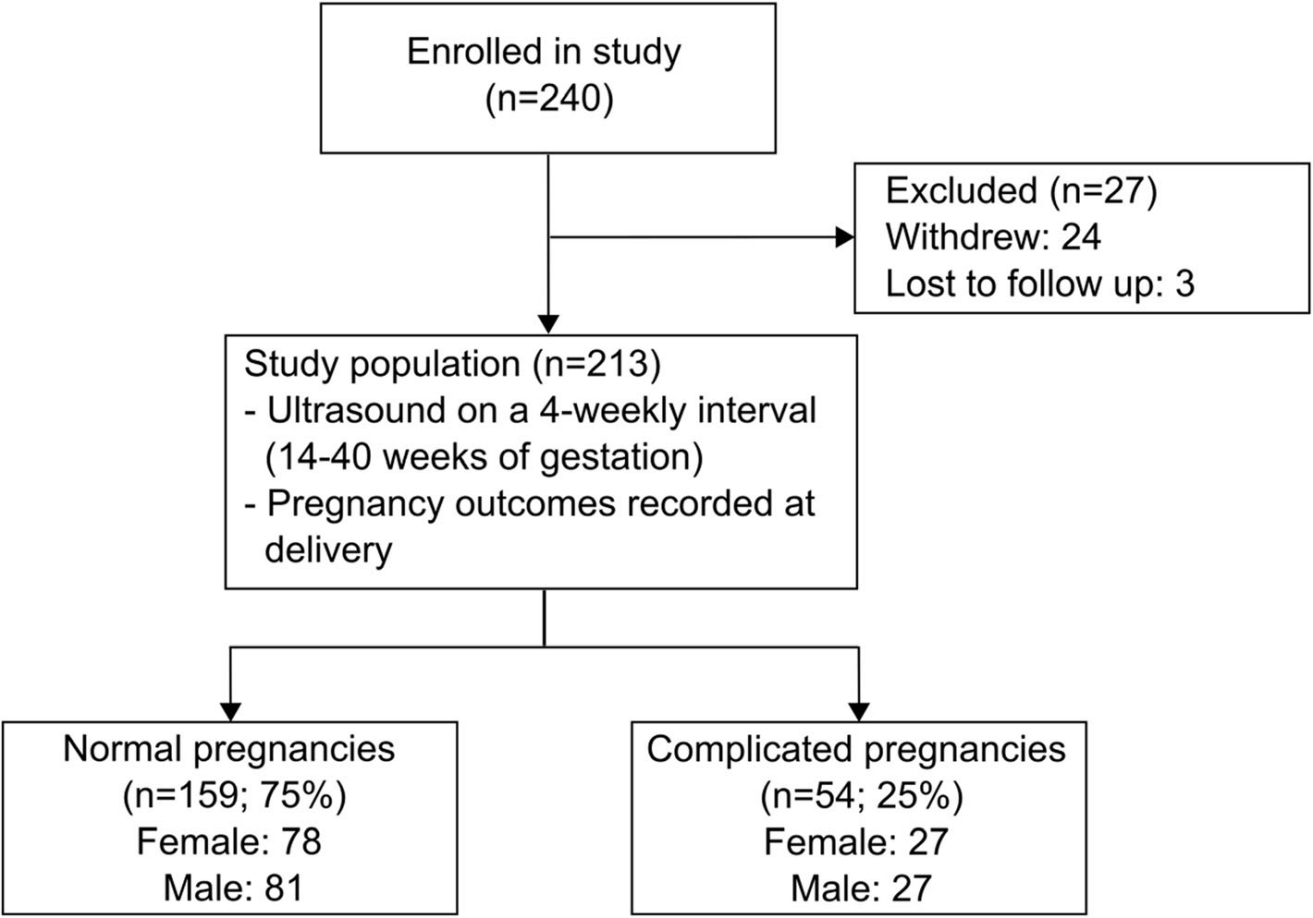
Diagram of flow of participants through the study. Complicated pregnancy defined as one or more of maternal preeclampsia, preterm birth, or small for gestational age

Figure 1 summarizes the number of participants at each stage of the study. Of the 240 women who consented to participate in the study, 213 women underwent ultrasound examinations throughout gestation (24 withdrew and 3 delivered at another site and were lost to follow up). The 213 participants provided a total of 1140 uterine artery measurements (average of 5 per participant). One hundred and fifty-nine of the 213 women (75%) met the criteria for the normal pregnancy group (78 female and 81 male infants) and 54 (25%) met the criteria for the complicated pregnancy group (27 female and 27 male infants). The clinical characteristics of these participants are summarized in Table 1. The birth weights in complicated pregnancies were significantly lower than the normal group (*p* < 0.0001), while male infants weighed more than females in both groups (*p* < 0.001). In complicated pregnancies, there was no significant effect of fetal sex on the incidence of preeclampsia (22% females and 30% males), preterm birth (48% females and 56% males), or SGA (44% females and 44% males).

**Table 1:** Comparison of maternal demographics and pregnancy and neonatal outcomes

Characteristic	Normal Pregnancy		Complicated Pregnancy	
	Female (*n* = 78)	Male (*n* = 81)	Female (*n* = 27)	Male (*n* = 27)
Maternal age at delivery (years)	35 ± 4	34 ± 4	33 ± 3	35 ± 4
Pre-pregnancy BMI (kg/m^2^)	25 ± 5	25 ± 5	26 ± 6	27 ± 7
Race (%)				
Asian	25 (20/78)	19 (15/81)	26 (7/27)	30 (8/27)
Black or African American	8 (6/78)	12 (10/81)	22 (6/27)	22 (6/27)
White	63 (49/78)	58 (47/81)	45 (12/27)	45 (12/27)
Other	4 (3/78)	11 (9/81)	7 (2/27)	3 (1/27)
Cesarean delivery (%)	35 (27/78)	40 (32/81)	44 (12/27)	56 (15/27)
Gestational age at delivery (weeks)	38 ± 1	39 ± 1	37 ± 2^a^	36 ± 3^a^
Birth weight (g)	3200 ± 400	3500 ± 500^b^	2500 ± 700^a^	2600 ± 700^a,b^

^a^*p* < 0.05 when compared to normal pregnancy

^b^*p* < 0.05 when compared to females

Data are mean ± standard deviation or % (n/N)

*BMI* body mass index; *SGA* small for gestational age

### Uterine artery PI differed between normal and complicated pregnancies

**Fig. 2:**
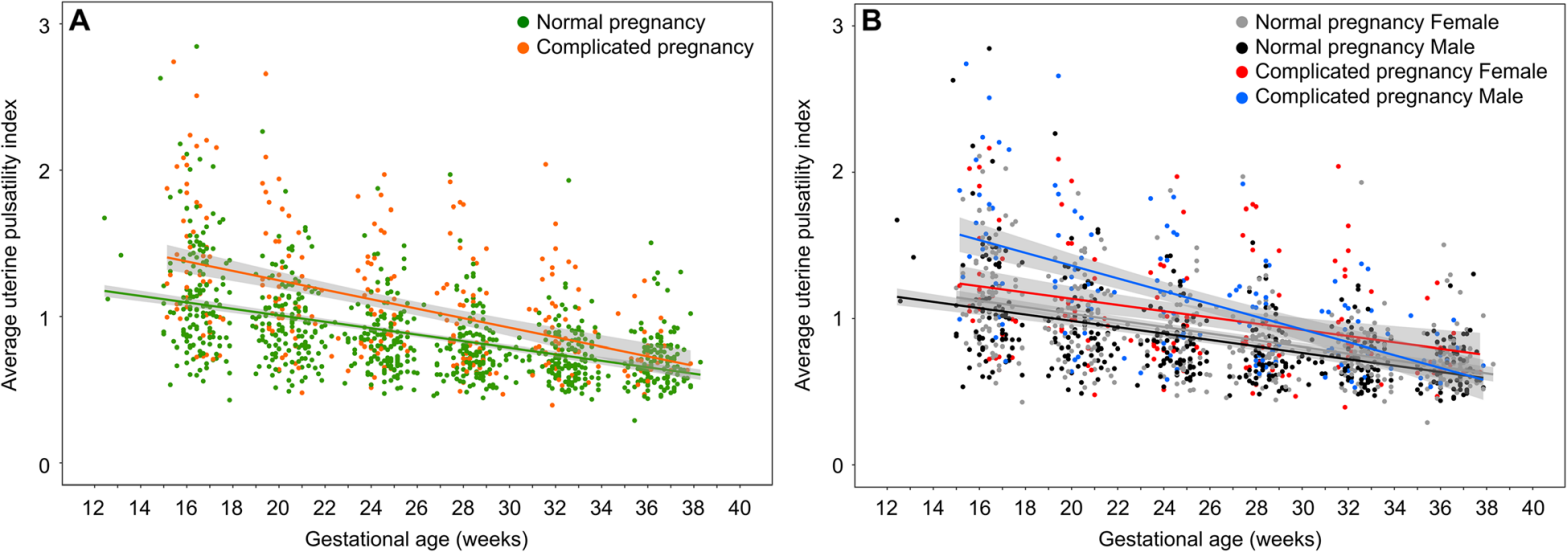
Average uterine artery pulsatility index over gestation. **a** Between the normal (green) and complicated pregnancies (orange), there were significant differences in the uterine artery pulsatility index (*p* < 0.0001) and in the rate of change in the uterine artery pulsatility index with gestation (*p* = 0.0003). **b** Between the normal (gray: female, black: male) and complicated pregnancies (red: female, blue: male), there were significant gestational age-related sex differences (*p* < 0.0001). The change in uterine artery pulsatility index over gestation was estimated by the slope of the regression. The male complicated pregnancy group had a higher slope of the line (−0.044 ± 0.005) compared to the other groups (complicated pregnancy female: −0.021 ± 0.005, normal pregnancy male: −0.022 ± 0.002, normal pregnancy female: −0.023 ± 0.002) (*p* < 0.0001). The shaded gray areas represent 95% confidence intervals

Figure 2a shows the change in average uterine artery PI over gestation for normal and complicated pregnancies. As expected, the uterine artery PI decreased with gestational age (*p* < 0.0001) and was significantly elevated in the complicated pregnancies compared to the normal pregnancies (*p* < 0.0001). The rate of change in the uterine artery PI with gestation was significantly different between the normal and complicated pregnancies (*p* = 0.0003). There was no effect of race on the uterine artery PI or the trajectory of the uterine artery PI with gestation.

### Trajectory of uterine artery PI differed by fetal sex for complicated pregnancies

Figure 2b shows the change in average uterine artery PI over gestation for the four study groups. The difference in the rate of change in the uterine artery PI with gestation between the normal and complicated pregnancies depended on fetal sex (*p* < 0.0001). Uterine artery PI change with gestational age was estimated by the intercept and slope of the regression. The male fetuses in the complicated pregnancy group had an elevated slope compared to the other groups (*p* < 0.0001), indicating a significantly greater change in uterine artery PI over gestation. Earlier in gestation (< 28 weeks), the uterine artery PI for male fetuses in complicated pregnancies was higher than in normal pregnancy. By term, the uterine artery PI for males in complicated pregnancies had returned to normal values. In contrast, the uterine artery PI for females in complicated pregnancies was elevated compared to normal pregnancies throughout gestation and, unlike the male complicated pregnancies, remains elevated at term.

## Discussion

In the present study, the uterine artery PI did not differ over gestation between females and males for normal healthy pregnancies, while it was dependent on fetal sex for pregnancies complicated by either preeclampsia, preterm birth, or fetal growth restriction. Our result in normal pregnancies is consistent with previous studies that reported no sex differences in uterine artery PI at 22-24 weeks’ gestation [26] and 31-37 week’s gestation [33]. A recent study by Broere-Brown et al. [32] found higher uterine artery PI (reported as a gestational-age-adjusted Z-score) in male fetuses from normal pregnancies in both the second and third trimesters. They also studied pregnancies complicated by preeclampsia, preterm birth, and fetal growth restriction and found the same trend, with males having higher uterine artery PI. Compared to the present study where we did not observe sex differences in normal pregnancies, the difference might be explained by the fact that while the Broere-Brown study had a larger number of participants (*n* = 4574), they only collected measurements at two-time points (once at 18-22 weeks’ gestation and once at 28-32 weeks’ gestation). Our study had a smaller sample size (*n* = 213); however, we obtained on average five measurements per participant, allowing us to accurately characterize the change in uterine artery PI over gestation.

An elevated uterine artery PI is associated with an increased impedance to flow in the uteroplacental circulation. In complicated pregnancies, females and males may have contrasting underlying placental pathologies, which deteriorate differentially, and are reflected in the difference in uterine artery PI values over gestation in abnormal pregnancy. Abnormal uterine artery blood flow early in gestation, consistent with the higher uterine artery PI values observed in complicated male pregnancies in this study, may partially explain the higher rates of stillbirth and the increased risk for preterm birth in male fetuses [10, 11, 34, 35]. The significantly higher rate of change in the uterine artery PI with gestation in males is in line with evidence that male fetuses grow faster than females, necessitating more significant maternal adaptations to increase blood flow to the placental circulation [36]. The lower uterine artery PI found here in females in the second trimester relative to males, could protect the pregnancy from early-onset complications. One adverse pregnancy outcome associated with female fetuses is a higher incidence of early-onset preeclampsia (< 34 weeks’ gestation) [16–18]. The uterine artery PI is typically measured during the first trimester and is found to be predictive of early-onset preeclampsia, with a lower success at identifying late-onset preeclampsia [37]. However, recent data has shown that assessment of impedance in the uteroplacental circulation by measuring the uterine artery PI during the third trimester has improved screening efficiency for late-onset preeclampsia [31, 38].

A limitation of the current study is that the sample size was underpowered to detect known sex differences in pregnancy outcomes (e.g., incidence of preeclampsia, mortality during gestation, and early neonatal death). Therefore, we were unable to investigate associations between sex-specific pregnancy complications and uterine artery PI. In addition, a larger sample size would allow us to separate the groups into different complications associated with impaired uteroplacental blood flow (e.g., preeclampsia vs. preterm birth). Another limitation of this study is that we were not able to capture uterine artery measurements during the first trimester.

## Perspectives and significance

The principal finding of this work is that changes in uterine artery PI over gestation in complicated pregnancies are dependent on fetal sex. This finding adds to the growing body of known sex differences in fetal and placental development. The uterine artery PI is widely used to assess uteroplacental function in clinical settings. Future investigations are needed to understand the underlying mechanisms for these sex differences in uteroplacental hemodynamics and if sex-specific reference ranges for uterine artery PI could improve pregnancy outcomes.

## Data Availability

The datasets used and/or analyzed during the current study are available from the corresponding author on reasonable request.
